# Hemophilia Gene Therapy: Approaching the First Licensed Product

**DOI:** 10.1097/HS9.0000000000000540

**Published:** 2021-02-10

**Authors:** Paul Batty, David Lillicrap

**Affiliations:** Department of Pathology & Molecular Medicine, Richardson Laboratory, Queen’s University, Kingston, Ontario, Canada.

## Abstract

The clinical potential of hemophilia gene therapy has now been pursued for the past 30 years, and there is a realistic expectation that this goal will be achieved within the next couple of years with the licensing of a gene therapy product. While recent late phase clinical trials of hemophilia gene therapy have shown promising results, there remain a number of issues that require further attention with regard to both efficacy and safety of this therapeutic approach. In this review, we present information relating to the current status of the field and focus attention on the unanswered questions for hemophilia gene therapy and the future challenges that need to be overcome to enable the widespread application of this treatment paradigm.

## Background

Since the cloning of the factor VIII (FVIII)^1,2^ and factor IX (FIX)^3,4^ genes in the mid 1980s, the goal to translate this knowledge into a clinically beneficial outcome has been progressively accomplished. First, with the use of molecular diagnosis for hemophilia,^5^ soon to be followed by the generation of recombinant clotting factor concentrates for replacement therapy.^6^ More recently, since 2011, the use of molecular genetic information has successfully been applied to deliver human hemophilia gene therapy.^7^

From the start of this journey, it was apparent that the development of successful gene therapy strategies for the 2 forms of hemophilia would face different challenges. In the very early days of hemophilia gene therapy investigations, George Brownlee (one of the scientists that cloned FIX) commented upon the profound lack of industry interest in engaging in studies of FIX gene transfer. Thirty years later, the recent announcement of the $450 million purchase of the UniQure FIX gene therapy program by CSL could be regarded as one obvious metric of success in the field.^8^

In contrast to FIX gene therapy, the subject of the first successful human hemophilia clinical trial in 2011,^7^ the path to success of FVIII gene transfer has been predictably more problematic. With a cDNA size 6-fold larger than FIX and a longstanding reputation for being problematic for molecular study, the FVIII gene transfer field has lagged behind FIX until the last 2 years. Now, at the end of 2020, it is unclear whether the first-licensed hemophilia gene therapy product will be for hemophilia A or B.

In this review, we will introduce the requisite elements of a hemophilia gene therapy program, briefly summarize the outcome of recent clinical trials, and discuss in more detail the many remaining unanswered questions as gene therapy edges closer to entering the clinic as a licensed hemophilia treatment.

## Transgene expression cassettes

As alluded to above, progress with the development of effective FIX gene therapy constructs has always surpassed that of FVIII expression cassette generation. The 1.5 kb FIX cDNA is easily packaged into a range of viral vectors, with expression mediated by liver-specific regulatory elements targeting the native site of FIX production. In addition, the discovery of a gain-of-function FIX variant^9^ (FIX Padua) has further enhanced the potential for attaining therapeutic FIX activity levels with moderate vector doses.^10^ This Arg338Leu missense variant found in FIX Padua increases the specific activity of the molecule approximately 7-fold, and extensive animal,^11^ as well as initial human studies,^10^ have shown no evidence of increased immunogenicity linked to this variant.

For FVIII, expression cassette development has been far more challenging. The size of the native FVIII cDNA of ~9 kb precludes packaging into clinically applicable vectors, and thus all current FVIII transgene constructs utilize a B domain-deleted (or truncated) cDNA. This process of using a truncated form of FVIII has previously found successful application to improve secretion from recombinant cell lines in FVIII concentrate manufacture without inducing increased immunogenicity. In a similar manner, replacement of the FVIII B domain with a 17 amino acid peptide containing 6 glycosylation sequences has also been demonstrated to enhance FVIII trafficking and secretion.^12^

As with FIX gene transfer, the target cell for FVIII gene therapy is the hepatocyte which, in contrast to FIX, is not the native cell of FVIII synthesis.^13,14^ This difference in the cell of origin of transgenic FVIII may explain, at least in part, the functional assay discrepancies that have been repeatedly observed, with 1 stage FVIII levels being ~1.6-fold higher than chromogenic FVIII values. A similar 1-stage versus chromogenic assay discrepancy has also been documented for FIX Padua, although it appears that the mechanisms underlying these discrepant results may be distinct. For hepatocyte-derived transgenic FVIII, the discrepancy appears to be associated with the earlier activation of factor X and subsequent faster generation of thrombin, although total thrombin levels are not affected.^15^ For FIX Padua, 1-stage FIX levels are also higher than chromogenic values, but in this instance, the levels of FX in the chromogenic assay reagent mix may be limiting FIXa detection.^16^

The final process that is now included in the generation of most expression cassettes involves codon optimization of the coding sequence to eliminate putative splicing sequences and maximize mRNA translation potential by matching the tRNA abundance in the intended transduced host cell.^17^ This process is most often performed using proprietary in silico algorithms.

## Transgene vectors

In 2020, recombinant adeno-associated viral vectors (AAV) are the predominant transgene delivery vehicle being used in hemophilia clinical gene therapy studies, an approach thought to be largely nonintegrating into the host genome.^18^ In the past, 1 hemophilia patient received an adenoviral vector infusion, there has been a trial of ex vivo electroporation of autologous fibroblasts^19^ and an in vivo study with a gamma retroviral vector.^20^ None of these alternate forms of transgene delivery showed sufficient benefit to merit further study. In addition to the various AAV trials, there is also an interest in utilizing lentiviral vectors as an alternative for patients with pre-existing neutralizing anti-AAV antibodies. Additionally, the integrating nature of lentiviral vectors could facilitate treatment in replicating cell types, for example, in the growing livers of children who could potentially benefit most from this treatment.^21^

With all this said, there is no doubt that the first series of licensed hemophilia gene therapy products will all be AAV-based, using a range of capsid serotypes, including some that have proprietary novel features. In all instances, the goal is to deliver the therapeutic transgene to hepatocytes following a single peripheral intravenous infusion. Clinical studies have indicated that this approach is effective in delivering sufficient vector to hepatocytes to result in therapeutic levels of clotting factor, although this process is still very inefficient.

Large-scale AAV production has recently seen significant advances with 2 strategies using either mammalian or insect cell cultures to manufacture the vectors.^22^ In addition to providing large-scale production to satisfy anticipated increasing patient demand, this has resulted in improvements in quality control ensuring better consistency with regards to vector genome packaging and a reduction in vector contaminants.^22^ While the optimal ratio of “full” versus “empty” vector particles remains unclear, most vectors are now generated with the intention of attaining a high capacity of “full” particles.

Two other vector variables bear further consideration: vector dose and vector serotype.

**Figure 1.**
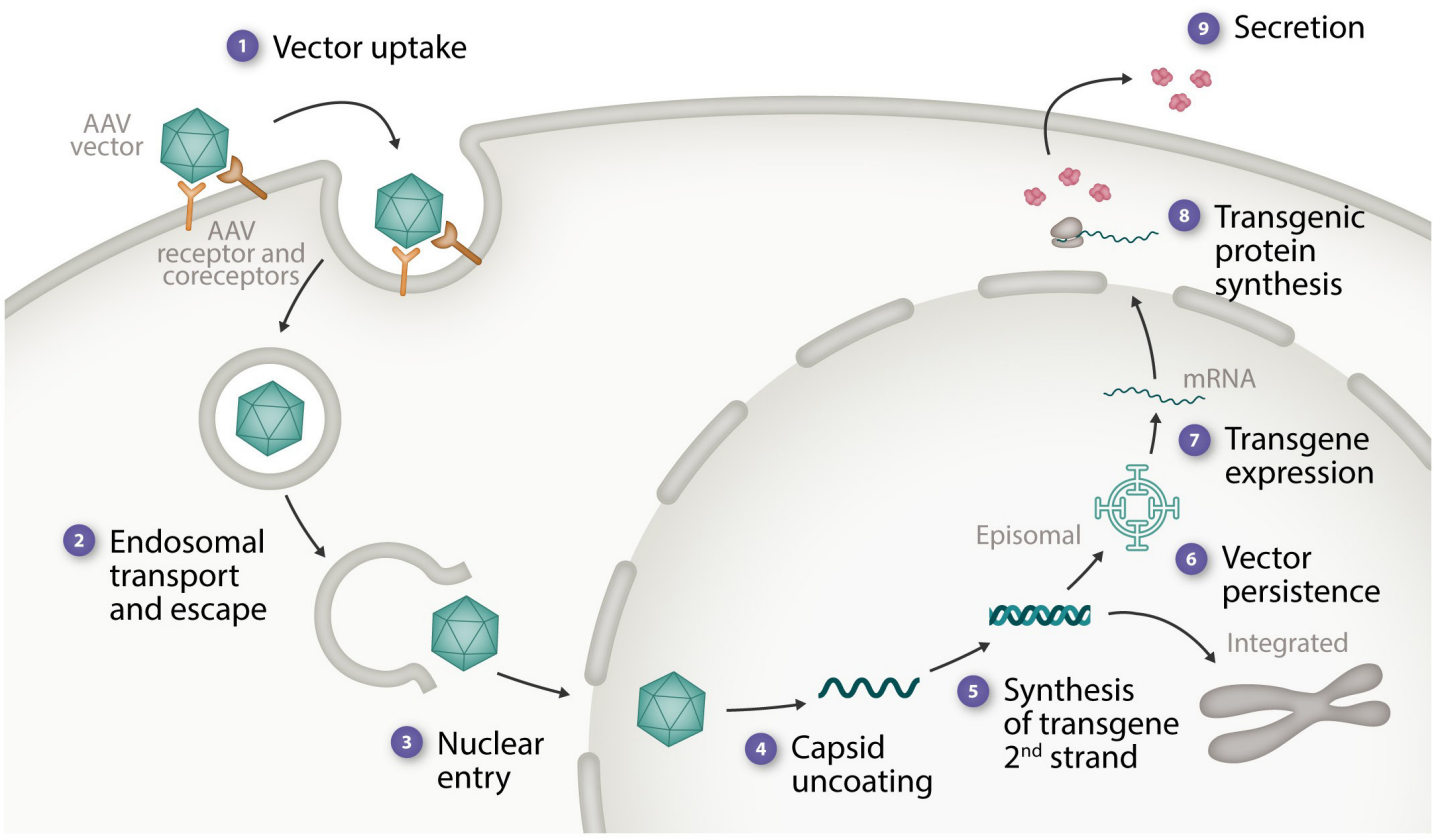
**Elements in AAV vector delivery and expression that likely contribute to variable transgene expression levels.** The stages in AAV vector delivery and transgene expression are listed along with some of the potential factors contributing to variable outcomes. (1) Vector uptake—role neutralizing anti-AAV Abs, numbers of glycan receptors, the AAV receptor, and coreceptors. (2) Endosomal transport and escape. (3) Nuclear entry—resting vs replicating cells, facilitated vs passive entry. (4) Vector capsid uncoating—speed, duration, and efficiency. (5) Synthesis of transgene second strand. (6) Vector persistence—% episomal, % integrated, influence of concatemer formation, genomic location of integrated copies. (7) Transgene expression—variable transcriptional silencing and activation (stress response elements), epigenetic remodeling of the transgene. (8) Transgenic protein biosynthesis—ER stress response, aggregate formation, and variable posttranslational modification. (9) Transgenic protein secretion and clearance—interaction with a carrier protein and clearance receptors. AAV = adeno-associated viral vectors.

Clinical hemophilia gene therapy studies have seen AAV vector doses that show a 300-fold range, from 2e11/vector genomes (vg)/kg^7^ to 6e13 vg/kg.^23^ These studies also involve many other variables (eg, vector serotype, vector production cell type, expression cassette differences), and, thus, it is remarkable and unclear why such a large range of vector doses ultimately result in relatively similar clotting factor protein levels. These outcomes highlight the complex multistep process of transgene delivery and expression (Figure 1) and also emphasize our profound ignorance about the details of most of these processes.

The last vector related detail to consider is the capsid serotype. Although there is knowledge of tissue tropism for most AAV serotypes, the precise details of how the targeted cells are transduced are still incomplete. In hemophilia gene therapy protocols, AAV serotypes that are hepatotropic have been used—AAV2, AAV5, AAV6, AAV8, and AAV10—as well as AAV vectors that have undergone proprietary capsid alterations but remain closely related to one of these native serotypes. With the many variables involved in gene therapy delivery and transgene expression, it is not obvious that any of these serotypes is clearly superior for hepatocyte delivery, and thus one other pragmatic reason for choosing a specific serotype concerns the pre-existing anti-AAV immune status of the patient.^24^

Neutralizing antibodies to AAV are present in 30%–80% of the general population with some differences between AAV serotypes within different ethnic groups and geographic locales.^25–27^ Of relevance for persons with hemophilia, 1 small study has suggested increased prevalence of AAV8 neutralizing antibodies in patients who have previously received plasma replacement therapy.^28^ The presence of these antibodies will usually inhibit successful transduction by the infused vector. Pre-existing immunity to AAV5, that is, the most distantly related AAV serotype, appears to be the least prevalent, and there is some evidence that even if anti-AAV5 antibodies are detected these may be of low affinity and will not interfere with hepatocyte transduction. There is also evidence that the type of pre-existing AAV antibody detected (neutralizing versus binding)^29^ and the presence of nonantibody inhibitory factors in plasma can interfere with AAV transduction^30^ thus further highlighting the complexity of this key area of AAV delivery efficiency.

The 1 hemophilia gene therapy program that currently differs significantly from the growing number of AAV studies is the recently opened phase 1 trial of platelet-derived FVIII gene therapy in hemophilia A patients with FVIII inhibitors.^31^ This program, centered at the Medical College of Wisconsin in Milwaukee, utilizes autologous CD34+ peripheral blood stem cells transduced ex vivo with a FVIII lentiviral vector and the application of a reduced intensity conditioning regimen to facilitate stem cell engraftment.

## Hemophilia gene therapy outcome measures

A critical element in any gene therapy protocol is the incorporation of a program of pragmatic and robust outcome measures. For hemophilia, this issue has been significantly aided by the analysis of clinical outcomes (musculoskeletal health and bleed rates) following usage of prophylactic clotting factor protein replacement therapy for the past 3 decades. In the laboratory, we can accurately measure plasma levels of FVIII and FIX. In the clinic, we can obtain patient-derived histories of bleeding events and can assess the impact on patients’ quality of life through validated questionnaires.^32,33^ Indeed, 1 might imagine that an improvement of “global” quality of life would be one of the most important outcomes of successful hemophilia gene therapy.^34^ Furthermore, and of major relevance to hemophilia, where the long-term morbidity of chronic joint bleeding represents the most important disease-related pathology, we can monitor musculoskeletal status with longitudinal imaging studies (eg, ultrasound and MRI).^35,36^ Finally, given the high cost of replacement therapy, health economic outcome assessments can be made to allow comparison to other therapeutic modalities. Thus, a combinatorial approach for outcome assessment of hemophilia gene therapy is well founded and should be able to ensure that an appropriate level of outcome objectivity is maintained.

Although measurement of FVIII and FIX plasma levels provides a direct and accessible way to determine transgene expression, there are still details of these assessments that require consideration. Recent studies evaluating expression of the transgenic FVIII and FIX proteins have demonstrated discrepancy in standard clinical laboratory measurements of clotting activity dependent on the type of assay used—the routine 1-stage functional assay versus chromogenic functional assays.^37^ In the case of FIX gene therapy, this difference appears to be due to the enhanced mode of action of the Padua variant protein,^38^ while for FVIII, where 1-stage assay results are ~1.6-fold higher than chromogenic results, faster activation of factor X, and earlier thrombin generation has been demonstrated in the 1-stage assay.^39^ Although the mechanism responsible for this outcome is unclear, it is possible that this results from alterations of posttranslational modification due to the use of a nonnative cell for protein production.^15^ These assay discrepancies suggest that further study is required to determine which assay result represents the “true” transgene derived plasma procoagulant activity. To allow transparency and comparisons in reporting, most studies will currently report the data from both assays.

Results of markedly reduced annualized bleed rates (ABRs) and reduced requirement of exogenous factor infusions have now been documented in several hemophilia gene therapy trials and accompanying measures of quality of life have shown a corresponding improvement. What has yet to be documented, because most trials are still of only 3–4 years duration, is any longer-term benefit in joint health and structure as determined by imaging analysis.

## Ongoing hemophilia gene therapy trials

In the fall of 2020, we seem likely to be 1–2 years away from the first licensed hemophilia gene therapy product. The Biomarin FVIII gene therapy program has recently undergone an initial assessment of their phase 3 trial results at the FDA, and this trial will be re-evaluated again toward the end of 2021. Close behind this initiative, the UniQure FIX gene therapy program will be approaching regulatory agencies for approval likely in 2021.

**Figure 2.**
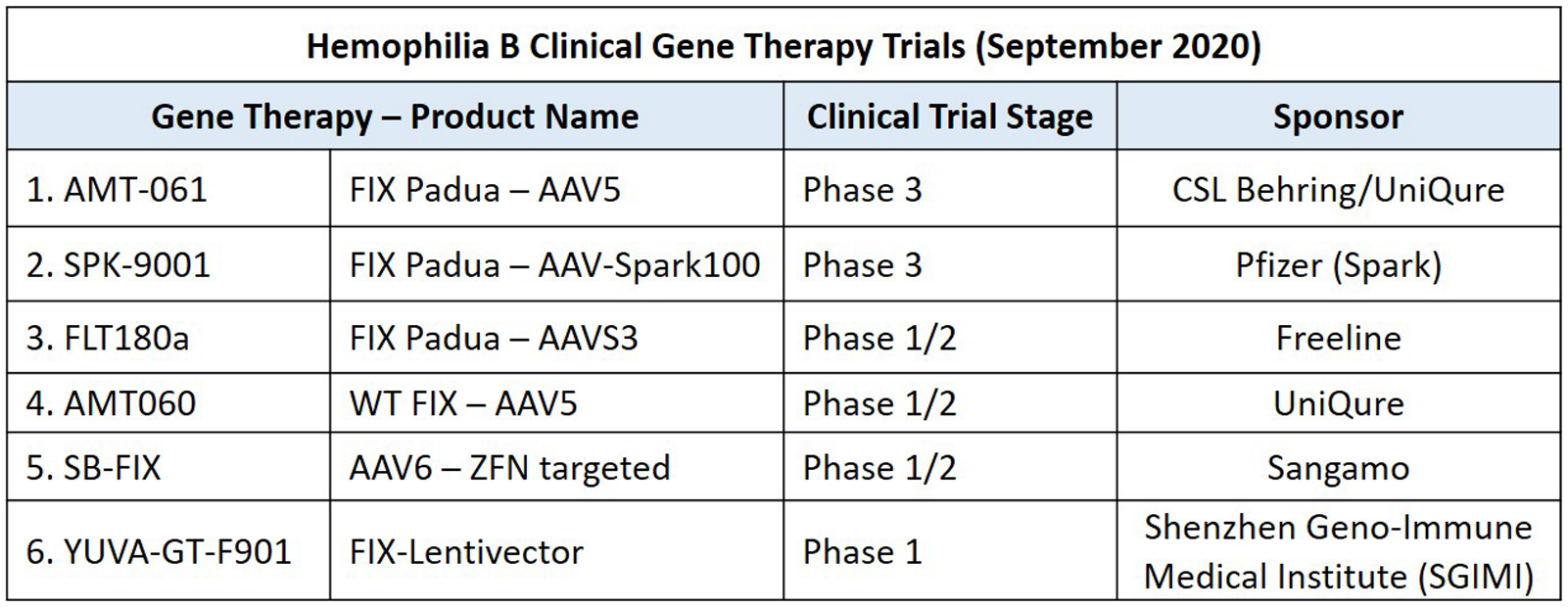
**Hemophilia B Clinical Gene Therapy Trials (September 2020).** Listed are the name of the gene therapy product, some of the vector details (eg, AAV vector serotype, form of FIX cDNA, and type of vector), the phase of clinical trial development and the industry sponsor of the study. AAV = adeno-associated viral vectors; FIX = factor IX.

**Figure 3.**
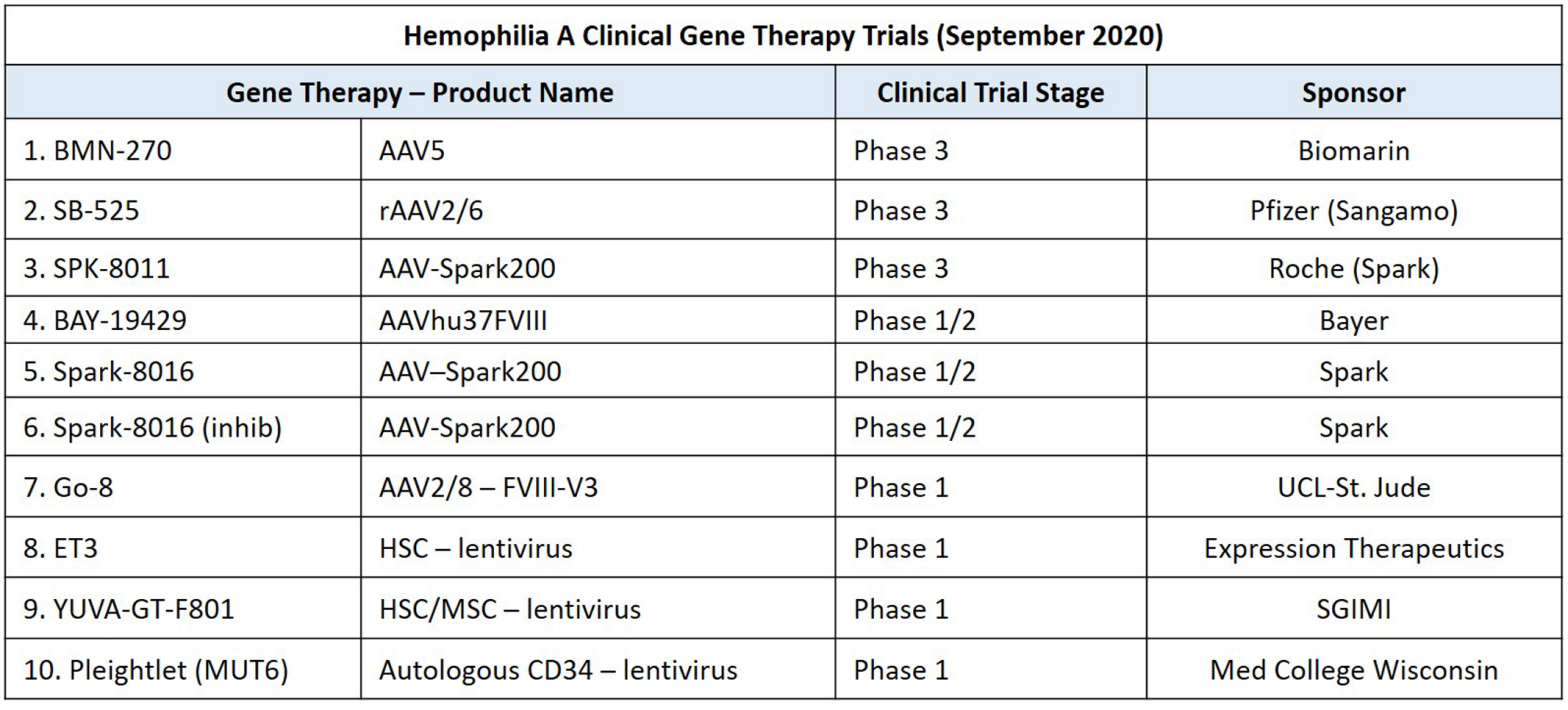
**Hemophilia A Clinical Gene Therapy Trials (September 2020).** Listed are the name of the gene therapy product and some of the vector details (eg, AAV vector serotype and type of vector AAV vs lentivirus), host cell type for cell-based gene therapy (eg, HSC or MSC), the phase of clinical trial development and the industry sponsor of the trial. AAV = adeno-associated viral vectors; HSC = hematopoietic stem cell; MSC = mesenchymal stem cell.

At this time, there is considerable activity in this gene therapy space. A review of the clinicaltrials.gov website in August 2020 documents 10 hemophilia A and 6 hemophilia B trials that are either still recruiting or remain active but no longer recruiting (Figures 2 and 3). We can expect that the first licensed hemophilia gene therapy products will be in clinics within the next couple of years, and the uptake of this new treatment will then depend upon a complex combination of payment options, patient satisfaction with current therapies, and ongoing uncertainties surrounding long-term gene therapy outcomes.

### Remaining unresolved challenges for hemophilia gene therapy

While progress with the clinical introduction of hemophilia gene therapy has been significant in recent years, there remain many unanswered questions about this radically new form of treatment. In the text below, we will summarize the current status of these ongoing challenges.

## Pre-existing immunity to AAV

Wild-type AAV infections in humans are asymptomatic and replication of AAV requires coinfection with a helper virus that is usually either adenovirus or herpes virus. Despite the lack of clinically recognizable infection, immunologic confirmation of prior exposure to AAV can be demonstrated in 30%–80% of subjects depending upon the AAV serotype, age, gender, and geographical location.^25–27,40,41^ It has been shown that in most instances, the identification of anti-AAV antibodies, even at low titer, will be sufficient to significantly impair therapeutically useful AAV vector delivery. This observation represents a clear obstacle to the wider application of AAV-mediated gene transfer. However, of note, 1 group using AAV5-mediated FIX gene transfer has shown that successful transgene expression can be accomplished despite the presence of pre-existing anti-AAV5 antibodies.^42^ While the definitive explanation for this achievement remains to be clarified it appears that this may relate to the fact that at least some of these anti-AAV5 antibodies have a low affinity for binding antigen and thus do not substantially interfere with vector delivery. Whether this finding can be replicated in other AAV5 studies or with other AAV serotype vectors remains to be seen.

Pre-existing immunity to AAV currently eliminates ~50% of patients who would otherwise be eligible for this treatment. Thus, in 2020, there remains a critical need to explore other strategies for clotting factor transgene delivery. One possible approach concerns the potential for significantly reducing the titers of anti-AAV antibodies with the infusion of an IgG cleaving enzyme (see text below re. IdeS). In the meantime, the most advanced alternative delivery approach for gene therapy involves lentiviral vectors that are now being evaluated for both systemic in vivo and ex vivo application in preclinical models.^21^ These integrating vectors have an ample packaging capacity for FVIII and FIX, and extensive preclinical and clinical assessment suggests that their patterns of genomic integration are very unlikely to result in oncogenic transformations.

Aside from recombinant viral vector protocols, progress has also been made using nonviral lipid-based nanoparticle delivery, although the efficiency of delivery and attainment of long-term transgene expression remains some way from clinical application.^43^

## Potential for AAV vector readministration

Following systemic delivery of the quadrillions of AAV vector particles applied in gene therapy protocols, there is an inevitable production of a very robust and long-lived anti-AAV capsid immune response. The outcome of this profound immune reactivity, as seen in large animal models and clinical studies,^44,45^ is that more than 10 years following vector delivery, these antibodies are likely to neutralize any readministered vector. Furthermore, cross reactivity with other vector serotypes is usually sufficient to negate any benefit that might accompany switching vector serotypes. The consequence of these findings is that current AAV delivery is viewed as a “one chance only” therapeutic opportunity.

The potential importance of AAV vector readministration is regarded as a high priority in the field and thus major efforts are being made to mitigate this obstacle using a variety of immune-modulatory strategies. One recent novel strategy for overcoming this obstacle is the use of the IgG endopeptidase, Imlifidase (IdeS). In preclinical evaluations of this agent in both mouse and nonhuman primate models of AAV vector immunity, IdeS significantly enhanced vector delivery to the liver in the context of pre-existing anti-AAV neutralizing antibodies.^46^ These preliminary preclinical results suggest that this strategy might be an important advance to overcome the limitations posed by pre-existing AAV immunity and for the purpose of AAV vector readministration.

## Early transient liver toxicity

While acute adverse events following AAV vector delivery are rare, there is an incidence of liver toxicity that occurs in ~60% of patients between 4 and 12 weeks postvector delivery.^7,23^ This complication is marked by usually mild/moderate increases in hepatocyte-derived serum alanine aminotransaminase levels, a fall or loss of expression in the plasma level of the transgenic clotting protein (due to death of transduced hepatocytes), and in some patients, evidence of AAV capsid-specific cytotoxic T cells (by positive interferon gamma ELISpot assays).^47^

The pathogenesis of the liver toxicity remains unclear and may indeed be different in different patients. The 3 mechanisms that appear most plausible and have support from experimental investigation are (a) an anti-AAV capsid peptide cytotoxic T cell response,^47^ (b) a result of endoplasmic reticulum stress and subsequent hepatocyte apoptosis due to high clotting factor expression^48^ (much more likely with FVIII transgenes), and (c) a direct effect of vector particle load.

While the pathogenetic details of liver toxicity require further clarification as a matter of high priority, empiric therapies are being used to mitigate this problem. In most cases, this amounts to oral corticosteroid therapy for 2–3 months at an initial starting dose of ~1 mg/kg and then dose reducing. However, in occasional instances, the hepatotoxicity is unresponsive to oral steroids and has required the administration of intravenous methylprednisolone or alternative immune regulatory agents (eg, Tacrolimus or azathioprine). Finally, where liver toxicity has been encountered more frequently in initial studies, a prophylactic schedule of oral steroids has been used.

The growing dilemma related to liver toxicity is that we are still unsure about the pathogenic mechanism(s) and the use of increasingly complicated and potentially harmful immunosuppressive treatment regimens is a cause for concern. This issue has now been further highlighted with the recent reports of 3 deaths from progressive liver dysfunction in the ASPIRO study, investigating a high-dose AAV8 therapy for X-linked myotubular myopathy.^49^ Although all 3 patients had pre-existing hepatobiliary disease and the vector doses used in this study were 5-fold higher (3e14vg/kg) than is currently used in hemophilia gene therapy studies, more basic knowledge of causation is urgently required. As these changes are not seen following vector delivery in animal models, the attainment of this knowledge will very likely require access to human liver biopsy samples at the time of the liver injury for detailed histopathological and molecular examination.

## Predictability of transgene expression level and durability

Probably the most critical questions that patients will ask about potential outcomes of hemophilia gene therapy are (a) what level of factor will I achieve with gene therapy? and (b) how long will the treatment last? In 2020, the answer to the second of these questions can be addressed with reference to both large animal^50^ and human study data,^51^ but the question of predictability of the eventual clotting factor levels remains unanswerable at this time.

In terms of durability of transgene expression, human FIX gene therapy studies in adult patients are now out to 8 years post-single intravenous administration and have shown only minimal evidence of a decline in plasma FIX levels. Similarly, studies in the canine model of hemophilia A have demonstrated persistent therapeutic expression of FVIII of over a decade following AAV vector infusions.^50^ Somewhat in contrast, the results of the longest duration human FVIII gene transfer trial using an AAV5 vector has shown a significant decline in FVIII levels over the first 4 years postvector delivery. Levels of FVIII are now around 0.20 IU/mL,^52^ a level that is still producing substantial bleed protection and we await with keen interest to see if these levels now stabilize or continue to fall. Thus, collectively these results all suggest that AAV vector delivery in adult humans and dogs accomplishes therapeutic clotting factor expression for at least several years and in the hemophilic dog model for periods greater than a decade. Definitive clinical data requires at least 5 years of follow up to make confident statements concerning the long-term durability of transgene expression.

While the evidence to support statements concerning AAV gene transfer durability is helpful, we have almost no information to provide to patients concerning their individual plasma level of transgenic protein. In human trials to date, there is significant variability in these levels that in some instances is as high as >10-fold (0.20 to >2.00 IU/mL).^52^ It should be remembered that even levels of native FVIII and FIX have a normal population variability of 4-fold and that the balance and complexity of the production, secretion, and clearance of these proteins are still only partially understood.

In hemophilia gene therapy, this complexity and the number of unknown facts is significantly increased. We have very little information available concerning details of hepatocyte entry, trafficking within the hepatocyte, the efficiency of nuclear import, and the process by which the transgene remains episomal or integrates into the host-cell genome (Figure 1). Similarly, details of posttranslational trafficking and modification, the interaction with other plasma proteins (particularly the FVIII-VWF interaction) and transgenic protein clearance mechanisms are lacking. The only conclusion that can reasonably be made is that we need much more basic research into the details of AAV vector biology and that, in the meantime, the attainment of specific clotting factor levels is totally unpredictable.

## Long-term safety and genotoxicity

A theoretical safety advantage of AAV vector delivery is the fact that this delivery system does not routinely result in the integration of vector sequences into the host genome, thus reducing the risk of long-term insertional oncogenicity. Nevertheless, there has been evidence in studies involving recombinant AAV gene transfer into neonatal mouse models that hepatocellular carcinoma can develop,^53,54^ and fragmented wild-type AAV genomes have been found in liver cancers.^55^ However, whether AAV gene transfer is associated with an enhanced genotoxic risk for oncogenicity remains unknown.^56^ Of note, detailed autopsy and histopathological examination of hemophilic dogs that have lived for >10 years following AAV vector delivery has not documented any evidence of malignant tumors in the liver.^57^

Very recently, liver biopsy material from humans treated with AAV vectors has been obtained and evaluated for the presence and genomic forms of AAV persistence.^58^ Furthermore, liver biopsies from long-term AAV treated hemophilic dogs have also undergone extensive genomic analysis^57,59^ While the results of these preliminary studies will require further confirmation, several features of AAV vector persistence in the liver have been robustly documented, these include (a) that most (>95%) of AAV vector copies persist even after a decade, as episomal, nonintegrated forms (Figure 1), (b) there are substantial numbers of AAV vector integrations in liver cell genomes, occurring with a frequency of between ~1 per 1,000 and 10,000 cells, (c) >90% of these integrated vector copies are in intergenic regions of the genome, and finally (d) there is at least some nonrandom location of the integrated vector copies with some genomic sites being prone to repeated vector insertions.^57,59^

As with our lack of understanding concerning the variability of transgene expression levels, more detailed information is also required to adequately explain the fate of vector genomes in the nucleus. Several key questions remain unanswered—what determines whether these sequences remain episomal in nature or become integrated into the host genome, is what appears to a semiselective process for vector integration predictable based on details of the host genome structure, and where does long-term transgene expression derive from—episomal or integrated vector copies? Long term follow-up will be of importance to determine whether there are rare unexpected adverse events. Current recommendation from the FDA suggests 5 years follow-up after AAV treatment and an international hemophilia gene therapy registry is being developed to enable longer-term vigilance.^60^

## The potential of hemophilia gene therapy in children

All hemophilia gene therapy trials to date have involved heavily pretreated adult patients, and it is reasonable to ask whether the recent successes documented in these studies could be extended to a pediatric population. This is an especially pertinent question at a time when AAV-mediated gene transfer is being used in very young children with severe inherited neurological conditions.^61,62^

The major challenge for using AAV vector delivery in children is that because of the predominantly nonintegrating nature of these vectors, a major proportion of the vector could be lost from dividing cells during the substantial liver enlargement that occurs during childhood. However, despite the fact that this is a theoretical concern, we do not have direct evidence that this will happen. The recent finding of persistent episomal vector copies in the liver cells and many integrated vector copies >10 years after AAV delivery in hemophilic dogs suggests that this issue is far from resolved.^57^

Further preclinical investigation of this issue is urgently required as the application of gene therapy with sustained therapeutic factor levels would have the most benefit in children to prevent recurrent musculoskeletal bleeding and its associated long-term morbidity.

## The potential of hemophilia gene therapy in inhibitor patients

Another patient group that has been excluded from hemophilia gene therapy trials so far are those with current and past histories of FVIII and FIX alloantibodies (inhibitors). This treatment-related complication occurring in ~30% of severe hemophilia A and ~3% of severe hemophilia B patients is a major challenge to the effective hemostatic management of these patients.^63^ While the recent introduction of the bispecific monoclonal antibody, emicizumab for use in FVIII inhibitor patients represents a significant advance in the management of these patients, there is still a sense that regaining immunologic tolerance to FVIII, to allow a reintroduction of FVIII treatment in these patients, is preferable. For the infrequent patients with FIX inhibitors, the situation is far more challenging and complex, with no obviously superior treatment strategy.^64^ Attempts at tolerance induction in these patients are often associated with an immune complex-associated nephrotic syndrome and anaphylaxis, and, ultimately, these patients may require long-term management with rFVIIa infusions.

Given the very significant negative influence of inhibitor development on hemophilia care, the potential intervention with gene therapy is a reasonable consideration, and, indeed, in the dog model of hemophilia A, there is objective evidence that FVIII tolerance can be achieved through AAV-mediated gene transfer to the liver.^65^

Looking ahead, it seems that small-scale clinical studies might be conducted on FVIII inhibitor patients who have failed conventional immune tolerance induction (ITI) attempts and who are anxious to be rid of their inhibitors. These patients can still be treated with emicizumab to prevent bleeding in the interim, and, following gene therapy, if emicizumab use is still required in the early weeks following vector delivery, transgene expression can be followed effectively with the use of a chromogenic FVIII assay employing bovine reagent components.

This application of gene therapy would rely upon 2 features that cannot be achieved with current ITI protocols: the attainment of sustained, persistent levels of antigen (FVIII) delivery and the expression of the antigen in the tolerogenic environment of the liver.

## Payment options for hemophilia gene therapy

Much has been discussed in recent years about the question of how much a potentially curative intervention such as gene therapy should cost, and once a cost is established how this is paid.^66,67^ Although this issue will continue to be contentious, at least for hemophilia, there are already very well documented cost standards for long-term care in which >95% of the expense derives from the therapeutic product.^68,69^ Based on this knowledge, a justifiable market value for hemophilia gene therapy could be proposed to be derived from annual clotting factor costs for a schedule of prophylactic therapy, with an additional up-front supplement to take into account product development costs. Subsequent to vector delivery, a necessary but more complicated formula will need to be established to incorporate the therapeutic performance over time, with presumably termination of payments if transgenic clotting factor levels fall below a therapeutically relevant minimum. This will require the careful development of costing models involving industry members, clinicians, patient advocates, and health economists. Given the major differences in funding for hemophilia care around the world, it is likely that different countries will have different payment schema, similar to that which currently occurs with tender processes for coagulation factor concentrates. The coincident emergence of other innovative strategies for the treatment of hemophilia and the cost reduction of factor replacement therapies brought about by tender processes will further complicate this issue.

The issue of gene therapy costs not only presents a major challenge in developed countries but poses an even greater financial and ethical dilemma in developing countries, where this therapeutic modality has the most potential to make a major positive impact.^70^ How these critical but very complex questions will be resolved awaits the imminent arrival of the first licensed product for at least an initial glimpse of how much this treatment will cost.

## Summary and conclusions

It is now >35 years since the cloning of the FVIII and FIX genes, and, during this time, major advances have been made in the application of molecular genetic knowledge to hemophilia diagnostic testing and in the generation of novel bioengineered recombinant clotting factor concentrates. There is no doubt that these advances have substantially enhanced the safety and efficacy of hemophilia clinical care and have improved the quality of life of this patient population.

The application of molecular biology knowledge to the field of gene therapy has also occupied the minds of many biomedical scientists for a similar length of time, but in contrast to the advances witnessed in diagnostic testing and recombinant protein innovation, evidence of clinical benefit for the use of gene therapy has been limited and relatively recent.

There now seems little doubt that the first hemophilia gene therapy product will be approved for clinical use within the next 2 years, an outcome that reflects the safety profile and clinical benefit achieved in current, ongoing phase 3 studies. This advance obviously represents an immensely important milestone in hemophilia management, and the patient community should be appropriately excited by the potential of this development. Similarly, hemophilia treaters should also be very enthusiastic about the arrival of a treatment option that has the potential for major long-term benefits. However, the availability of this new treatment paradigm will require treaters and patients alike to give considerable thought as to who best to consider for this significant intervention, given the number of other novel therapeutics that will be available at this time.

Finally, although the time for clinical hemophilia gene therapy appears to be close, there remain many unanswered questions that will impact the outcome of this treatment. It is critical that further basic and preclinical investigation of AAV gene transfer continues as we try to better understand the details of this complex process.

## Disclosures

DL receives research support from Bayer, BioMarin, CSL, Octapharma, and Sanofi. PB has received research support from BioMarin, Grifols, and Octapharma.

## Acknowledgments

DL is the recipient of a Canada Research Chair in Molecular Hemostasis. PB is the recipient of a Queen’s University SEAMO Clinical Research Fellowship Award. The authors’ hemophilia gene therapy research is supported in part by a CIHR Foundation Grant (FDN 154285) and by a grant from the Canadian Hemophilia Society.
